# Normative Data on Platelet Count, Mean Platelet Volume, Platelet Distribution Width, Platelet-Large Cell Ratio, and Plateletcrit in Neonates

**DOI:** 10.7759/cureus.89293

**Published:** 2025-08-03

**Authors:** Jawairia Jabeen, Sunanda Jha, Varun Garg, Shambhavi Datta

**Affiliations:** 1 Department of Pediatrics, Rajendra Institute of Medical Sciences, Ranchi, IND

**Keywords:** gestational age, neonates, platelet indices, thrombocytopenia, total platelet count

## Abstract

Introduction

Platelet indices, encompassing mean platelet volume (MPV), platelet distribution width (PDW), platelet-large cell ratio (P-LCR), and plateletcrit (PCT), along with platelet count, are fundamental for assessing the hematological health of neonates. In neonatal populations, especially within the first 28 days of life, establishing normative data on these indices is essential, as neonatal platelet physiology differs markedly from that of older children and adults. Elevated MPV can reflect an active bone marrow response to platelet consumption, while increased PDW may signify platelet anisocytosis and variation due to bone marrow stress. P-LCR provides insight into the proportion of large, young platelets, which may indicate hematological stress or recovery. PCT, representing total platelet volume, offers a comprehensive measure of overall platelet mass.

Methodology

This cross-sectional observational study was conducted at a tertiary care hospital to establish normative reference values for platelet counts and indices in neonates from 0 to 28 days. The study cohort included neonates admitted to the Neonatal Intensive Care Unit (NICU), Special Newborn Care Unit (SNCU), and postnatal wards. Platelet count and platelet indices, specifically MPV, PDW, P-LCR, and PCT, were measured using a standardized automated hematology analyzer (Yumizen H2500, Horiba, Montpellier, France) to ensure consistency and accuracy. Blood samples were collected following neonatal venipuncture protocols to minimize any pre-analytical variations that might affect platelet measurements. Stratified analysis was conducted by gestational age, birth weight, and postnatal age to provide age and condition-specific reference ranges. Descriptive statistics and percentile-based reference intervals were employed to define normative data for each parameter.

Results

The study analyzed a total of 999 neonatal samples, with 435 female samples and 564 male samples. The mean total platelet count (TPC) was found to be 243,059.3 ± 90,741.3 per mm³, with a reference range (5th to 95th percentile) of 135,000-430,000 per mm³. The mean MPV was 9.5 ± 1.4 fL, with a range of 7.2-12.1 fL. PDW showed a mean of 14.3 ± 4.5 fL and ranged from 8.0 to 21.9 fL. The P-LCR had a mean of 26.6 ± 8.7% with a reference range of 14.6-42.2%, and PCT averaged at 0.23 ± 0.09% within a 0.12-0.39% range. Gestational age and postnatal age had no significant correlation with TPC or platelet indices. There were no significant differences in these platelet parameters based on gender except for P-LCR. TPC negatively correlated with PDW (r = -0.132, p = 0.001) and P-LCR (r = -0.092, p = 0.01) while showing a strong positive correlation with PCT (r = 0.879, p < .001). MPV positively correlated with P-LCR (r = 0.715, p < .001) and PCT (r = 0.295, p < .001), underscoring interdependencies among platelet indices in neonatal blood profiles.

Conclusion

This study provides comprehensive normative data on platelet count and indices (MPV, PDW, P-LCR, and PCT) in neonates within the first 28 days of life. The reference ranges established here can serve as critical benchmarks for neonatal hematological assessment, offering clinicians reliable parameters for interpreting platelet profiles in neonates.

## Introduction

Platelets are small, anucleate fragments derived from megakaryocytes, primarily involved in thrombus formation and primary hemostasis. Over the past decades, their functional scope has expanded beyond clotting, as they are now recognized for their roles in inflammation, angiogenesis, immune defense, and tissue repair through the release of over 300 bioactive proteins and interaction with endothelial and immune cells [1,2]. These diverse functions highlight their critical role in maintaining vascular integrity and modulating immune responses. Despite their importance, most platelet research has historically centered on adult physiology, leaving a significant gap in our understanding of platelet behavior in neonates, particularly during the first 28 days of life, a period marked by rapid hematological and systemic adaptation.

Neonatal platelets are known to exhibit functional hyporesponsiveness when compared to adult counterparts. This includes diminished secretion of α-granules, reduced aggregation in response to thrombin and collagen, and lower surface expression of glycoproteins such as GPIIb/IIIa, particularly in preterm infants [3]. These structural and functional differences, while not necessarily pathological, underscore the need for population-specific reference values. The neonatal hemostatic system is not merely an immature version of the adult system. It is qualitatively distinct and uniquely adapted to the transitional physiology of early life.

The first 28 days of neonatal life encompass dramatic hematologic, vascular, and immunologic shifts, making this period critical for establishing accurate, age-specific normative values for platelet indices. Standard adult reference ranges often do not account for the physiological nuances of neonates, especially those born prematurely or exposed to perinatal complications. This mismatch can result in misinterpretation of laboratory data and unnecessary interventions. Among the various parameters measured by automated hematology analyzers, platelet indices serve as accessible, cost-effective tools for evaluating platelet morphology, activation status, and production kinetics.

Mean platelet volume (MPV) reflects the average size of circulating platelets and serves as a marker of platelet activation and turnover. Platelet distribution width (PDW) indicates size variability and increases with platelet activation, while plateletcrit (PCT), akin to hematocrit, represents total circulating platelet mass. The platelet-large cell ratio (P-LCR) quantifies the proportion of larger, often more reactive platelets, and may reflect increased thrombopoiesis or peripheral destruction [3,4]. Despite their clinical relevance, the utility of these indices in the neonatal population remains underexplored, and normative ranges specific to this age group are yet to be firmly established.

In addition to intrinsic developmental differences in megakaryopoiesis and platelet function, neonatal platelet parameters are influenced by a host of maternal and perinatal factors. Conditions such as maternal hypertension, intrauterine growth restriction, prenatal drug exposure (e.g., aspirin, magnesium sulfate), neonatal infections, and interventions like nitric oxide therapy or mechanical ventilation can lead to transient platelet dysfunction or fluctuations in count and morphology [3,4]. Term neonates who are small for gestational age and those with perinatal asphyxia frequently exhibit lower platelet counts with elevated MPV values, indicating increased platelet turnover under stress. Similar patterns have been observed in preterm neonates with respiratory distress, where thrombopoiesis may be amplified in response to peripheral destruction or inflammation [4,5]. These physiological and pathological influences further complicate the interpretation of platelet indices unless contextualized within neonatal-specific norms.

Establishing reliable reference intervals (RIs) for platelet parameters is therefore essential for accurate clinical assessment in neonates. These intervals assist in guiding transfusion decisions, monitoring disease progression, and identifying neonates at risk for bleeding or thrombosis. Yet, much of the currently available data is extrapolated from adult or older pediatric cohorts, which may not accurately reflect the hematologic state of neonates. Moreover, the rapid developmental trajectory during this period demands sex- and age-specific reference standards. This study aims to establish normative data for total platelet count and platelet indices, MPV, PDW, PCT, and P-LCR, in neonates from 0 to 28 days of life. It also explores their associations with gestational age and birth weight to better inform clinical practice and optimize neonatal outcomes.

## Materials and methods

This cross-sectional observational study was carried out in the Department of Pediatrics, Rajendra Institute of Medical Sciences (RIMS), from January 2023 to December 2023 following approval from the Institutional Ethics Committee, Rajendra Institute of Medical Sciences, Ranchi with memo no.78. Informed written consent was obtained from the parents of all participants at the time of recruitment into the study. The study included neonates delivered at RIMS, residing in the postnatal ward, and those admitted to the NICU (Neonatal Intensive Care Unit) and SNCU (Special Newborn Care Unit) at the Department of Neonatology, Rajendra Institute of Medical Sciences, Ranchi, Jharkhand.

Inclusion criteria

Neonates (24 to 42 weeks of gestation) admitted to the NICU/SNCU or the postnatal ward who were appropriate for gestational age (AGA) were included.

Exclusion criteria

Neonates who were small for gestational age (SGA) or large for gestational age (LGA) and with conditions affecting platelet count, including sepsis, hemolytic disease, congenital infections, aneuploidy, and moderate/severe perinatal asphyxia, were excluded. Additionally, babies born to mothers with a history of placental insufficiency, e.g., eclampsia/preeclampsia/hypertension/HELLP/platelet-lowering medications, were also excluded.

Convenience sampling was employed, with the sample size determined by the maximum number of neonates available to capture population variation, as formal calculations for normalizing laboratory parameters are not established. We were able to gather 999 sample reports throughout the year. Complete blood count (CBC) reports were collected from neonates, and platelet counts and indices were analyzed on a Yumizen H2500 (Horiba, Montpellier, France) with follow-up CBC reports recorded throughout the neonatal hospital stay. Neonates were segregated into preterm and term categories based on the expanded new Ballard scoring. To assess the variability of parameters across the first 28 days of life, reports were collected and categorized based on days of life (DOL) into DOL (1-3), DOL (4-7), and DOL (>7), but we could collect very few samples of >7 days of life.

Data was collected using a structured form, detailing birth information like mode of delivery and relevant risk factors along with age, sex, birth weight, and gestational age. Laboratory values for platelet counts and indices were compiled for neonates in the NICU/SNCU/postnatal ward. Data were analyzed using Microsoft Excel 2019 (Microsoft Corporation, Redmond, USA) and IBM SPSS Statistics for Windows, Version 20 (Released 2011; IBM Corp., Armonk, New York, United States), presenting qualitative data as proportions and quantitative data as means and standard deviations. Significance tests were conducted at a level of <0.05, employing the Mann-Whitney U test, Kruskal-Wallis test, and Spearman correlation coefficient to evaluate significant variable relationships.

Reference intervals

Unlike adults, neonates lack defined standard ranges due to ethical apprehensions associated with procuring blood samples from healthy neonates purely for research purposes. As a result, an alternative methodology, known as RIs, is employed. These intervals encompass values ranging from the 5th to the 95th percentile, derived from diagnostic tests conducted on specific neonates for clinical reasons. The process for selecting the data to be included in the reference range involved retrospectively identifying CBCs from neonates thought to have minimal disorders relevant to the laboratory test, or with disorders unlikely to significantly affect the test results. In order to accommodate the derivation of reference ranges from patients rather than healthy volunteers, a distinct convention was employed for these ranges in contrast to standard ranges. Standard ranges encompass 95% of recorded values, excluding the lowest and highest 2.5%. In contrast, reference ranges entail the inclusion of 90% of values while excluding the lowest and highest 5%, thereby delineating values falling between the 5th and 95th percentiles.

## Results

The demographic details in this cross-sectional study are listed in Table 1.

**Table 1:** Newborn demographics The data has been represented as "N" and "%"

Total newborns (n = 999)	N	%
Female	435	43.54
Male	564	56.46
Birth weight		
Normal birth weight (NBW = 2500 – 3999g)	562	56.26
Low birth weight (LBW = 1500 – 2499Gg)	355	35.54
Very low birth weight (VLBW = 1000 – 1499g)	71	7.10
Extremely low birth weight (ELBW = <1000g)	11	1.10
Gestational age		
Term (37 – 41weeks+6days)	526	52.65
Late preterm (34 – 36weeks+6days)	167	16.72
Moderate preterm (32 – 33weeks+6days)	210	21.02
Very preterm (28 – 31weeks+6days)	85	8.51
Post term (=42 weeks)	8	0.80
Extremely preterm (<28weeks)	3	0.30
Days of life		
1-3	808	80.88
4-7	157	15.72
>7	34	3.40
Mode of delivery		
Vaginal delivery	584	58.46
Cesarean section	415	41.54

This cross-sectional study analyzed 999 newborns, with a slight male predominance (56.5%, n=564) compared to female newborns (43.5%, n=435). Birth weight distribution revealed that 35.5% (n=355) were low birth weight (1500-2499g), 7.1% (n=71) very low birth weight (1000-1499g), and 1.1% (n=11) extremely low birth weight (<1000g), whereas the rest 56.3% (n=562) were normal birth weight (2500-3999g). Gestational age analysis showed 52.7% (n=526) term births (37-41 weeks+6 days), with preterm categories comprising 16.7% (n=167) late preterm (34-36 weeks+6 days), 21.0% (n=210) moderate preterm (32-33 weeks+6 days), 8.5% (n=85) very preterm (28-31 weeks+6 days), and 0.3% (n=3) extremely preterm (<28 weeks). The majority of samples (80.9%, n=808) were collected within the first three days of life, with 15.7% (n=157) at 4-7 days and 3.4% (n=34) beyond seven days. Regarding delivery mode, 58.5% (n=584) were vaginal births versus 41.5% (n=415) cesarean sections. 

**Figure 1:**
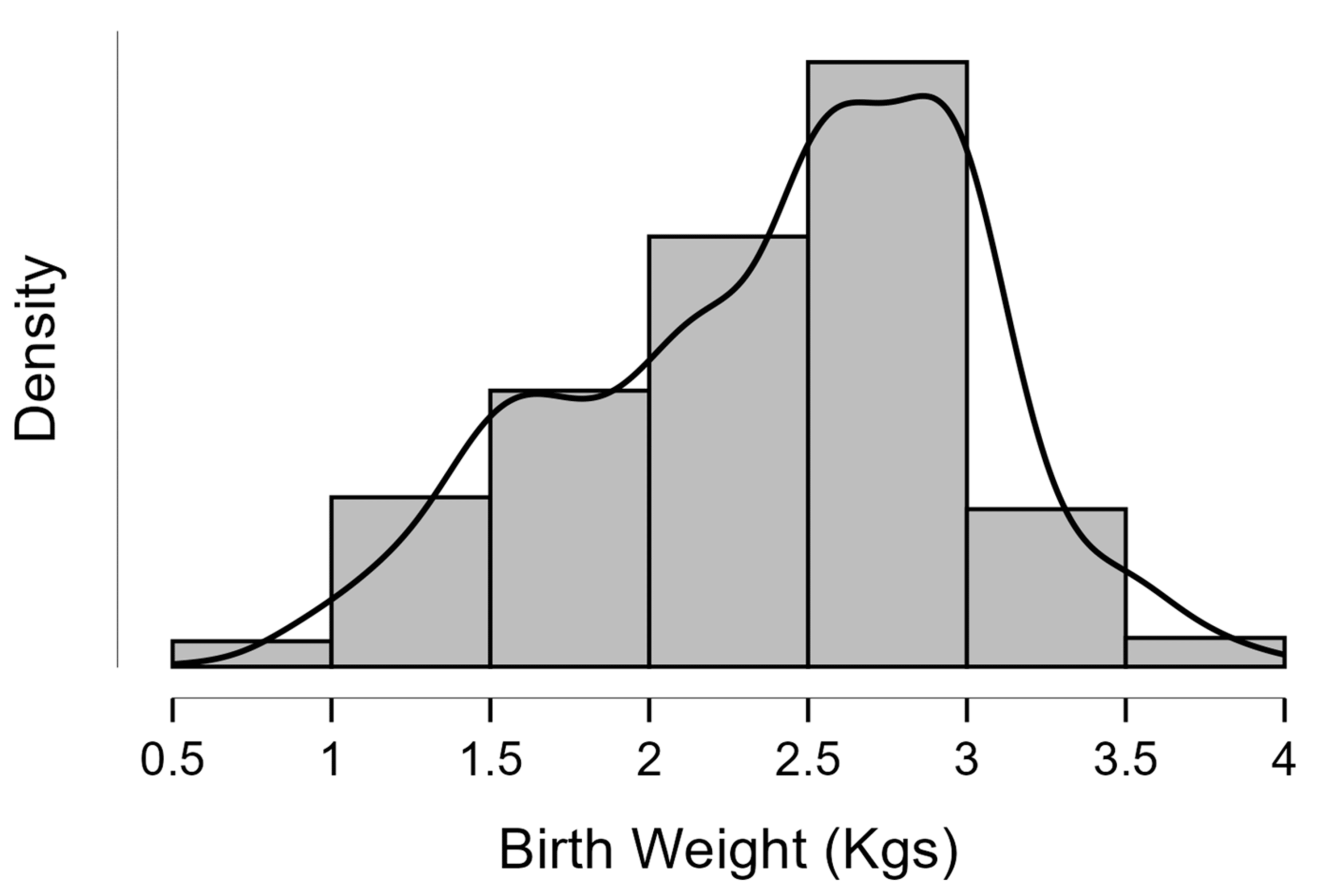
Distribution plot of birth weight Birth weight represented in kilograms (Kgs)

**Figure 2:**
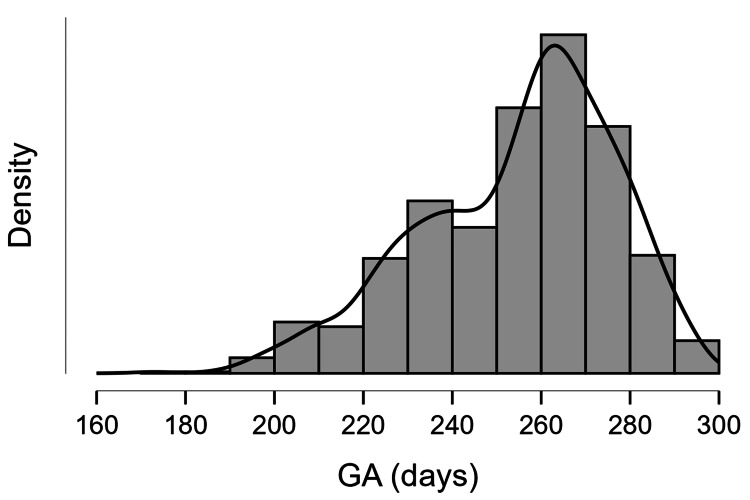
Distribution plot of gestational age Gestational age represented in days

The mean birth weight was 2.41±0.63 kgs (2.39±0.62 kgs for female neonates and 2.44±0.63 kgs for male neonates), and the mean gestational age was 254.91±21.77 days (254.02±21.45 days for female neonates and 255.59±22.0 days for male neonates). The distribution plot can be seen in Figures 1, 2, respectively.

Platelet counts and platelet index levels

The RIs for platelet parameters are mentioned in Table 2. 

**Table 2:** Reference intervals for platelet counts and platelet indices TPC: Total Platelet Count; MPV: Mean Platelet Volume; PDW: Platelet Distribution Width; P-LCR: Platelet Large Cell Ratio; PCT: Plateletcrit; fL: Femtolitre

Platelet parameters	5th percentile	95th percentile	Median	Mean	Std. Deviation
TPC (per mm^3^)	135000	430000	231000	243059.3	90741.28
MPV (fL)	7.2	12.1	9.4	9.477	1.44
PDW (fL)	8	21.9	14.7	14.268	4.455
P-LCR (%)	14.6	42.2	25.3	26.561	8.716
PCT (%)	0.12	0.391	0.22	0.23	0.092

Comparison between female and male babies

Female neonates had higher venous blood total platelet count (TPC) values (244 (±93) ×10^3/mm^3^) than male neonates (241 (±88) ×10^3/mm^3^), but this difference was not statistically significant (p value = 0.902) using the Mann-Whitney U test. Similarly, there was no significant difference found in the platelet indices among the male and female neonates, except for P-LCR (p value = 0.036), in which male neonates had significantly higher values than female neonates, as shown in Table 3.

**Table 3:** Mean platelet counts and indices of female and male neonates The platelet parameters are expressed in mean (SD) The Mann-Whitney U test was used p-value significant at <0.05

Platelet parameter	Female neonates	Male neonates	U-value	p value
TPC (per mm^3^)	244779.310(±93290.572)	241732.624(±88785.710)	123227.500	0.902
MPV (fL)	9.464(±1.415)	9.487(±1.460)	118726.500	0.383
PDW (fL)	14.050(±4.525)	14.436(±4.397)	115216.000	0.099
P-LCR (%)	25.992(±8.730)	26.999(±8.689)	113208.000	0.036
PCT (%)	0.232(±0.094)	0.229(±0.090)	123046.500	0.934

Comparison between term and preterm babies

As the post-term (42weeks) neonates were very few (n=8), those were excluded while comparing means between term and preterm neonates. It was found that there was no significant difference in platelet parameters between term and preterm neonates (Table 4) using the Mann-Whitney U test.

**Table 4:** Mean platelet counts and indices for term and preterm neonates The platelet parameters are expressed in mean (SD) The Mann-Whitney U test was used p-value significant at <0.05

Platelet parameters	Term	Preterm	U-value	p-value
TPC (per mm^3^)	245471.863(±90942.692)	240369.892(±89624.917)	127208.500	0.275
MPV (fL)	9.477(±1.502)	9.482(±1.367)	122377.500	0.985
PDW (fL)	14.295(±4.394)	14.277(±4.547)	123091.500	0.859
P-LCR (%)	26.654(±8.896)	26.486(±8.566)	122250.000	0.992
PCT (%)	0.231(±0.091)	0.229(±0.092)	123736.000	0.748

Comparison between NBW and LBW

MPV and P-LCR values were higher in low birth weight (LBW) and TPC, PDW, and PCT values were lower in LBW babies, but there was no significant difference in these parameters between normal and low birth weight neonates (Table 5).

**Table 5:** Mean platelet counts and indices for normal and low birth weight neonates NBW: 2500-3999 grams, LBW: <2500 grams Platelet parameters expressed in mean (SD) The Mann-Whitney U test was used p-value significant at <0.05

Platelet parameters	NBW (Normal birth weight)	LBW (Low birth weight)	U-value	p-value
TPC (per mm^3^)	246797.509(±91360.740)	238251.716(±89813.930)	130406.500	0.093
MPV (fL)	9.448(±1.499)	9.514(±1.361)	121006.000	0.692
PDW (fL)	14.273(±4.403)	14.261(±4.526)	123507.000	0.875
P-LCR (%)	26.446(±8.824)	26.708(±8.584)	118730.000	0.369
PCT (%)	0.232(±0.092)	0.228(±0.091)	125623.000	0.532

Comparison between vaginal mode of delivery and cesarean delivery

It was found that PDW values were significantly higher in caesarean deliveries as compared to vaginal mode, with a p-value of 0.025 as shown in Table 6 using the Mann-Whitney U test.

**Table 6:** Mean platelet counts and indices for vaginal and cesarean mode of delivery Platelet parameters expressed in mean (SD) The Mann-Whitney U test was used p-value significant at <0.05

Platelet parameters	Vaginal delivery	Cesarean delivery	U-value	p-value
TPC (per mm^3^)	244529.623(±93386.183)	240990.120(±86952.458)	121753.500	0.899
MPV (fL)	9.530(±1.434)	9.403(±1.447)	126989.000	0.196
PDW (fL)	14.053(±4.563)	14.570(±4.285)	111100.500	0.025
P-LCR (%)	26.348(±8.774)	26.860(±8.636)	117957.500	0.473
PCT (%)	0.233(±0.095)	0.226(±0.088)	125763.500	0.307

Comparison of the presence and absence of complications during delivery

There was no significant difference found in the platelet parameters between the presence or absence of complications during delivery, except for MPV values which were found to be significantly higher in neonates born to mothers with complications during delivery (like preterm labor, obstructed labor, cord prolapse, fetal distress, etc) as shown in Table 7 using the Mann-Whitney U test.

**Table 7:** Mean platelet counts and indices in the presence and absence of complications during delivery Platelet parameters expressed in mean (SD) The Mann-Whitney U test was used p-value significant at <0.05

Platelet parameters	No complications	Complications present	U-value	p-value
TPC (per mm^3^)	247114.610(±88837.715)	241251.664(±91582.721)	112614.500	0.141
MPV (fL)	9.244(±1.460)	9.581(±1.420)	92706.000	0.001
PDW (fL)	14.204(±3.579)	14.296(±4.796)	112355.500	0.158
P-LCR (%)	25.672(±7.909)	26.957(±9.030)	99433.500	0.097
PCT (%)	0.227(±0.091)	0.232(±0.092)	103138.500	0.436

Comparison between different days of life categories

Total platelet counts decreased as the days of life increased, but no statistically significant difference was found using the Kruskal-Wallis test. Similarly, there was no significant difference in platelet indices in subsequent days of life after birth (Table 8).

**Table 8:** Mean platelet counts and indices at different days of life DOL: Days of life Platelet parameters expressed in mean (SD) The Kruskal-Wallis test was used p-value significant at <0.05

Platelet parameters	DOL (1-3)	DOL (4-7)	DOL (>7)	H Statistic	p-value
TPC (per mm^3^)	243481.683(±89505.944)	241968.153(±94334.404)	238058.824(±104875.089	0.828	0.661
MPV (fL)	9.465(±1.425)	9.455(±1.509)	9.874(±1.561)	1.355	0.508
PDW (fL)	14.222(±4.489)	14.644(±4.101)	13.621(±5.172)	2.784	0.249
P-LCR (%)	25.489(±8.601)	26.752(±8.869)	27.376(±10.755)	0.285	0.867
PCT (%)	0.230(±0.092)	0.228(±0.092)	0.235(±0.097)	0.335	0.846

Correlation of birth weight, gestational age (days), DOL, TPC, MPV, PDW, P-LCR, and PCT

A Spearman correlation matrix was constructed to evaluate the relationships among neonatal demographic variables, birth weight, gestational age (GA), and days of life (DOL), and platelet indices, including TPC, MPV, PDW, P-LCR, and PCT. The results are detailed in Table 9.

**Table 9:** Correlation between different platelet parameters, birth weight, gestational age and days of life *P < 0.01 and **P < 0.001 (2-tailed); −: Negative correlation; +: Positive correlation; 1: No correlation; GA: Gestational age; DOL: Days of life; Spearman correlation was used

Variable	Birth Weight (Kgs)	GA (days)	DOL	TPC (per mm3)	MPV (fL)	PDW (fL)	P-LCR (%)	PCT (%)
Birth Weight (Kgs)	1	0.917**	-0.023	0.037	-0.041	-0.001	-0.039	-0.011
GA (days)	0.917**	1	-0.011	0.022	-0.028	-0.034	-0.038	-0.013
DOL	-0.023	-0.011	1	-0.026	0.005	0.027	0.012	-0.009
TPC (per mm^3^)	0.037	0.022	-0.026	1	-0.025	-0.132**	-0.092*	0.879**
MPV (fL)	-0.041	-0.028	0.005	-0.025	1	0.021	0.715**	0.295**
PDW (fL)	-0.001	-0.034	0.027	-0.132**	0.021	1	0.509**	-0.124
P-LCR (%)	-0.039	-0.038	0.012	-0.092*	0.715**	0.509**	1	0.152**
PCT (%)	-0.011	-0.013	-0.009	0.879**	0.295**	-0.124**	0.152**	1

A statistically significant and strong positive correlation was observed between birth weight and GA (r = 0.917, p < 0.001), reflecting the inherent physiological association between fetal growth and maturity. Neither birth weight nor GA demonstrated significant correlations with DOL or any of the platelet indices, suggesting that the hematological parameters studied are largely independent of these growth-related variables within the neonatal period. TPC exhibited a strong positive correlation with PCT (r = 0.879, p < 0.001), consistent with the fact that PCT represents the volume fraction of platelets in whole blood and is derived from both platelet count and volume. TPC also showed statistically significant but weak inverse correlations with PDW (r = -0.132, p < 0.001) and P-LCR (r = -0.092, p < 0.01), indicating that higher platelet counts may be associated with a narrower distribution of platelet size and a reduced proportion of large platelets. MPV was significantly and positively correlated with both P-LCR (r = 0.715, p < 0.001) and PCT (r = 0.295, p < 0.001), indicating that increases in average platelet size are associated with greater overall platelet mass and a higher proportion of large platelets. PDW also demonstrated a significant positive correlation with P-LCR (r = 0.509, p < 0.001), but an inverse correlation with PCT (r = -0.124, p < 0.001), suggesting that increased platelet size variability may not directly correspond to increased total platelet volume. DOL did not exhibit any statistically significant correlation with the platelet indices analyzed, implying that these hematological parameters remain relatively stable over the first 28 days of life in healthy neonates.

## Discussion

This study establishes normative data for five key platelet indices, TPC, PCT, MPV, PDW, and P-LCR, in neonates aged 0 to 28 days. Our findings offer a comprehensive reference framework critical for clinical and research applications, particularly in the early neonatal period, where hematologic values are dynamic and yet frequently used for diagnostic decision-making.

Our results demonstrated no significant correlation between gestational age and platelet counts. These findings are consistent with several previous reports. Hohlfeld et al. found the mean platelet count as 245 ± 65 × 10^3^/mm^3^ (similar to ours 243 ± 90 × 10^3^/mm^3^) without significant variation between 17 and 41 weeks' gestation [6]. Forestier et al. also found no effect of gestational age on platelet counts [7]. However, Wiedmeier et al. demonstrated that postnatal platelet counts do indeed increase with advancing gestational age [8]. The absence of significant correlations between DOL and any platelet indices suggests that these parameters remain relatively constant over the first four weeks of life in healthy neonates. This suggests that although some hematologic parameters in neonates are age-sensitive, platelet indices remain relatively stable throughout the first month of life. This is corroborated by Sakhno et al., who reported minimal variation in platelet values within the first three days of life, further validating the age-invariant utility of these indices during the neonatal period [9]. This observation is also consistent with Chang et al., who noted minimal variations in platelet indices across different postnatal age groups, implying the early establishment of platelet homeostasis [10].

Rolim et al. concluded that male newborns had lower platelet values than female newborns [11], which is quite opposite to that observed in our study, where there was no significant difference in platelet counts between male and female newborns. Newborns delivered by vaginal birth presented with higher platelet counts compared to those born by C-section, possibly due to the higher body water content in cesarean deliveries seen by Rolim et al. Still, we found no significant differences in platelet counts between the two modes of delivery [11]. We also found elevated PDW in neonates delivered via cesarean section, compared to those born vaginally, which may reflect subtle hematologic adaptations or stress responses during the birth process. PDW, which measures the variability in platelet size, can increase due to reactive thrombopoiesis or the release of immature platelets from bone marrow. Although most existing studies primarily focus on total platelet counts rather than indices of delivery mode, indirect evidence suggests that physiological stress during cesarean delivery could influence megakaryocyte activity, thereby affecting platelet morphology. Further research specifically examining PDW variations with delivery mode is warranted to clarify these associations.

The median and RIs observed in this cohort align with previously reported neonatal platelet norms; however, slight variations reflect population differences, methodology, and the timing of sample collection. Our values are comparable to those reported by Cui et al., who also derived RIs for platelet indices from capillary blood in healthy Chinese full-term neonates and observed relatively stable platelet counts across gestational and postnatal ages [12]. Similar observations were noted in the prospective study by Ianni et al., where platelet values in healthy term neonates at 24 hours of life were consistent with our results [13].

In our study, the mean TPC was 243 × 10³/mm³, which is lower than values reported by Cui et al. [12] (304 × 10³/mm³), Wasiluk et al. [14] (285 × 103/mm^3^) and Grecu et al. [15] (270 × 10³/mm³), but closely aligns with the findings of Sachdev et al. [16] (251 × 10³/mm³). This variation may be attributed to differences in the sample size, geographic population, and the timing of sample collection. MPV in our cohort was 9.48 fL, intermediate between the lower values reported by Wasiluk et al. (7.84 fL) and Grecu et al. (5.72 fL), and the higher MPV noted by Sachdev et al. (11.69 fL) and Cui et al. (10.9 fL), possibly reflecting differences in hematological adaptation or instrument calibration. For PDW, our study (14.27 fL) showed comparable values to that reported by Sachdev et al. (14.53 fL), although considerably lower than the extremely high PDW of 46.0 fL noted by Wasiluk et al., which could suggest methodological differences or outlier influence. The P-LCR in our neonates (26.56%) was lower than that reported by Sachdev et al. (38.44%) and the interquartile range reported by Cui et al. (25.0-34.7%), but significantly higher than that observed by Wasiluk et al. (6.26%), further highlighting potential technical or demographic influences. PCT was fairly consistent across studies, with our value (0.23%) aligning well with that by Wasiluk et al. (0.22%) and slightly lower than that by Cui et al. (0.30%) and Sachdev et al. (0.29%) [12,14-16]. These differences reinforce the need for population-specific reference ranges for neonatal platelet indices, considering variations in ethnicity, birth characteristics, and analytical platforms.

The comparative analysis of platelet parameters highlights the heterogeneity in standard reference values among various neonatal cohorts and methodological approaches, as illustrated in Table 10.

**Table 10:** Comparative analysis of various studies

Platelet parameters	Our study	Wasiluk et al. (2011) [14]	Sachdev et al. (2014) [16]	Grecu et al. (2014) [15]	Cui et al. (2020) [12]
TPC (×10^3^/mm^3^)	243±90	285.8±67.4	251±57.7	270±68	304±77
MPV (fL)	9.48±1.44	7.84±0.68	11.69±1.31	5.72±0.01	10.9 (10.1-11.4)
PDW (fL)	14.27±4.45	46.0±11.14	14.53±3.09	17.67±3.03	12.5 (11.1-15.8)
P-LCR (%)	26.56±8.72	6.26±3.94	38.44±10.82		30.0 (25.0-34.7)
PCT (%)	0.23±0.09	0.22±0.06	0.29±0.06	0.15±0.01	0.30 (0.26-0.36)

Interestingly, although we observed non-significant differences in platelet indices between neonates with normal and low birth weight, trends toward higher MPV and lower TPC and PCT in LBW neonates were apparent. These trends mirror those reported by Wasiluk et al., who documented lower platelet counts and altered indices in small-for-gestational-age neonates, likely due to intrauterine hypoxia or altered megakaryopoiesis [14]. The TPC demonstrated a strong positive correlation with PCT, which is expected, as PCT is a volumetric derivative of platelet count and size. This aligns with prior literature underscoring the close association between platelet mass and TPC in neonates [16]. Conversely, we found weak negative correlations between TPC and PDW as well as P-LCR, which may reflect a narrower size range and fewer large platelets in neonates with higher platelet counts, findings comparable to those by Wiedmeier et al., who observed stable counts in late preterm and term neonates, albeit with a broad range due to physiological variability [8]. Our analysis revealed a strong positive correlation between MPV and both P-LCR and PCT, indicating that an increase in the average platelet size is associated with a greater proportion of large platelets and overall platelet mass. This pattern aligns with the understanding that MPV reflects platelet production kinetics and can act as a proxy for megakaryocyte activity in the bone marrow. Similar associations have been reported by Wiedmeier et al., who found that MPV and P-LCR are interrelated measures indicative of platelet size heterogeneity and potential reactivity, particularly during the early neonatal period [8]. Additionally, Christensen et al. emphasized the relevance of MPV and related indices in reflecting the dynamic maturation of platelets in the neonatal circulation [17]. Moreover, a higher MPV in neonates born following complicated deliveries in our study echoes the findings by Rolim et al., who reported altered platelet parameters in neonates exposed to perinatal stress, highlighting the potential influence of perinatal complications on platelet activation [11].

In the context of platelet indices, Choudhary et al. observed that thrombocytopenia is inversely associated with MPV with established evidence suggesting that low platelet counts often trigger a compensatory response via increased production of larger, immature platelets by the bone marrow [18]. However, this inverse relationship was not statistically significant in our study. In a related study by Goyal et al., thrombocytopenia and elevated MPV and PDW were noted as significant predictors of neonatal sepsis, emphasizing their diagnostic utility in NICU settings [19]. A unique aspect of our study was the identification of a positive correlation between MPV and other platelet indices such as P-LCR and PCT in a cohort of healthy neonates. This association suggests that higher MPV values reflect increased platelet production and a greater proportion of larger platelets, which may indicate active megakaryopoiesis even in the absence of pathological stress. Some findings diverged from our observations like the JAMA Network study by Chen et al., which examined the impact of platelet transfusion on intraventricular hemorrhage and mortality and found that while thrombocytopenia was strongly associated with poor outcomes, the role of MPV was more ambiguous, with some models showing no independent prognostic value. Moreover, they reported that higher P-LCR values at the time of transfusion were not universally linked to increased risk, suggesting that P-LCR may not be as reliable a marker across all neonatal populations [20].

Taken together, our study reinforces the diagnostic and prognostic potential of platelet indices-particularly MPV, PDW, and P-LCR-in neonatal care, while also highlighting the need for cautious interpretation given the influence of multiple confounding variables, differences in analytical methods, and population-specific factors. Further multicenter, large-scale investigations with uniform methodologies are warranted to better define the utility of these indices across diverse neonatal subgroups.

Strengths and limitations

One of the primary strengths of this study lies in its large and diverse sample size of 999 healthy neonates, making the reference ranges for platelet indices more substantial and representative of the general neonatal population in the region. The inclusion of neonates across the full 0-28-day age spectrum, and stratification by relevant clinical parameters such as birth weight, gestational age, and mode of delivery, adds further depth to the findings. The use of standardized hematological analyzers and strict inclusion criteria-excluding neonates with infections, hematologic disorders, or other comorbidities-helped minimize potential confounding factors. However, the study has certain limitations. It was conducted at a single tertiary care center, which may limit the generalizability of the results to other settings with differing maternal and neonatal care practices. Additionally, although the study aimed to cover the entire neonatal period, the number of samples collected beyond seven days of life was relatively limited. This could have influenced the representativeness of the later age brackets. As this was a cross-sectional analysis, longitudinal trends in platelet indices over time could not be assessed. Inter-observer variability in sample collection and unmeasured environmental factors might also have influenced some of the hematological parameters, despite best efforts to standardize protocols. Lastly, although RIs were established, the study did not explore clinical outcomes or diagnostic utility in diseased neonates, which limits the immediate applicability of the findings in pathological contexts.

## Conclusions

This study establishes comprehensive normative data for platelet count and platelet indices, MPV, PDW, P-LCR, and PCT, in healthy neonates during the first 28 days of life. By evaluating a large and diverse cohort, we provide age-appropriate reference intervals that reflect the natural hematological variation across different gestational ages, birth weights, and modes of delivery. These findings can serve as a valuable reference for clinicians in differentiating physiological platelet variations from pathological conditions in neonatal practice. Although further multicenter and longitudinal studies are needed to validate these reference ranges and explore their clinical applicability, the present data lay important groundwork for improving diagnostic interpretation and guiding clinical decision-making in neonatal hematology.

## Appendices

**Table 11:** Case record form

Case No.	
Registration No.	
Name of neonate (B/O mother’s name)	
Father’s name	
Sex	
Birth weight	
Gestational age	
Days of life	
Mode of delivery	
Presence or absence of complications during delivery	
Total platelet count (/mm³)	
MPV (fL)	
PDW (fL)	
P-LCR (%)	
PCT (%)	
